# Pathological and Surgical Observations on the Diseases of the Joints

**Published:** 1841-04-03

**Authors:** 


### BIBLIOGRAPHICAL NOTICE.

*Pathological and Surgical Observations on the Diseases of the Joints.* By Sir Benjamin C. Brodie, Bart., F. R. S., Sergeant-Surgeon to the King, and Surgeon to St. George's Hospital. Fourth edition. London.

(Continued from page 155.)

We concluded our last notice of this work with the author's description of an *apparent* inflammation of the synovial membrane, which sometimes succeeds surgical operations:

"There is a peculiar morbid state of the system which in some instances follows severe accidents or operations, and which is well known to surgeons who are engaged in the practice of the London hospitals, in which the patients are liable to deposits of pus in various parts of the body at a distance from the seat of the original injury. These deposits not unfrequently take place in the cavities of the joints, as a consequence of inflammation of the synovial membrane, and independently of ulceration."

The nature of the affection is here clearly stated —it is a metastatic abscess of the joint, the index of a profound alteration of the blood; and we only regret that the author has entirely neglected to point out any means of distinguishing this from other articular swellings. The differential diagnosis is all-important; without it, an alteration of a joint, symptomatic of a general decomposition of the fluids, the immediate forerunner of almost certain death, may be confounded with simple rheumatism. We recal a case in point, where we witnessed this mistake on the part of one whose acumen in diagnosis cannot be called in question.

*Case.* A man named Vaddé, æt. 53, of strong constitution, entered the Hospital St. Louis, April 16, 1833. He presented a lacrymal tumour of the right side, about the size of a small filbert; this had existed for ten months. Pressure upon it failed to force out tears, either by the lacrymal points or by the nostril; the latter was habitually dry, and the eye wept incessantly. On the 18th the tumour opened spontaneously, but the epiphora continued. On the 20th, a silver canula was introduced with some difficulty. On the 21st, after venesection, the pain in the tumour was slight, the epiphora diminished, the nostril more moist, without, however, having given issue to any blood. On the 22d, the canula had risen and nearly escaped; it was forced back, and, an instant afterwards, blood issued from the right nostril. The canula having still a tendency to escape, was removed on the 25th, and, owing to the inflammation of the soft parts, was not replaced till May 12. At this date, the redness and tension had almost disappeared, and the epiphora had diminished; but a small amount of pus continued to exude from the opening, and the swelling of the soft parts remained so great that the straight tendon of the orbicularis could not be recognised; it was not till after numerous efforts that the nasal duct could be reached with a probe, which served to guide the canula; (an attempt to open it with a bistoury had proved unsuccessful.) Scarcely had the canula been introduced when it was forced out, and a pledget and bandage were found necessary to retain it in its place.

14*th.* The patient was attacked by a *chill*, succeeded by fever, with severe pain in right cheek and eye; this was followed by erysipelas.

18*th. Acute rheumatism* (?) of the left knee appeared during the night; the bowels were opened by an enema; 30 leeches and a poultice were applied to the knee; the amount of blood lost by the leeches produced syncope; the erysipelatous tint of the face disappeared entirely; the patient complained of general distress; the skin was cold, the pulse small and feeble. From this period the voice became more faint and embarrassed; there was no headach, and the intellect remained unimpaired.

20th. A painful œdematous swelling, unaccompanied by redness, occupied the back of the neck and the occipital region; in the evening the patient exhibited, for the first time, a derangement of intellect; this was followed on the 21st by low delirium—the prostration increased—the pulse continued small and feeble, but not very frequent. All the articulation became successively the seat of pain, and death occurred on the morning of the 22d.

The autopsy was made on the 23d. The membranes of the brain were slightly injected; the brain itself offered no alteration. The thoracic and abdominal viscera were healthy, with the exception of some large cicatrices of the mucous membrane of the greater curvature of the stomach near its pyloric extremity; over these points the membrane was whiter, more thin, but equally firm as elsewhere.

*Pus was found* in greater or less quantity in the articulations *of both knees*—in *the right elbow*—in *the left wrist*—and *in several of the toes*; in all the diseased articulations the synovial membrane was reddened and rather thickened; *the cartilages were unaltered.*

The mucous membrane of the nares was injected throughout their whole extent. The maxillary sinuses contained pus—the left lacrymal sac presented a redness of its lining membrane; the opening into the nasal duct of this side was large enough to permit the passage of the head of a pin. On the right side a fistula opened into the sac; the mucous membrane, of a grayish colour, was thickened and coated with purulent matter; at the inferior portion of the sac was found the head of the canula solidly fixed; the canula was directed obliquely backwards, and penetrated the nasal fossa opposite the middle turbinated bone; two-thirds of its length was exposed in the nostril.

The lower portion of the nasal duct was completely obliterated, and no trace of its inferior opening could be discovered; it could only be penetrated by removing the mucous membrane opposite the point at which it should have opened.

We have given this case not only as an illustration of the disease described by Sir B. Brodie, but particularly to call attention to its diagnostic signs. These are the same as in other metastatic abscesses. A *chill*, generally very severe, occurs within a few days after an operation. This is followed immediately by the symptoms of typhoid fever, and shortly afterwards several of the joints become very suddenly the seat of effusion; ordinarily the effusion attacks first the larger joints, but is rarely confined to them; depending upon a general cause, a purulent decomposition of the blood, it is not strange that joint after joint should be successively attacked, until the whole synovial system should participate in the common affection,—and this we find to be the case.

The author, in the present work, treats largely of ulceration of the articular cartilages. The possibility of this alteration is a question of such interest, that we defer its consideration to a future number.

